# Research Advances on Mesenchymal Stem Cell-Derived Exosomes in Anti-Graft-Versus-Host Disease Therapy: Mechanisms, Therapeutic Potential, and Future Prospects

**DOI:** 10.3390/ijms27093751

**Published:** 2026-04-23

**Authors:** Zihui Pan, Hui Wang, Qixiang Shao

**Affiliations:** 1 Jiangsu Key Laboratory of Medical Science and Laboratory Medicine, School of Medicine, Jiangsu University, Zhenjiang 212013, China; panzihui2026@163.com; 2 Institute of Medical Genetics and Reproductive Immunity, The Digestive and Reproductive System Cancers Precise Prevention Engineering Research Center of Jiangsu Province, School of Medical Science and Laboratory Medicine, Jiangsu College of Nursing, Huai’an 223005, China

**Keywords:** exosomes, graft-versus-host disease, mesenchymal stem cells, immunoregulation, Graft-versus-leukemia effect

## Abstract

Graft-versus-host disease (GVHD) remains the most severe complications following allogeneic hematopoietic cell transplantation (allo-HCT). Mesenchymal stromal cells (MSCs) have shown therapeutic potential in GVHD due to their immunomodulatory properties. However, their clinical application is constrained by safety concerns, including ectopic engraftment, microvascular obstruction, rejected by host, and potential tumor-supportive effects. Increasing evidence suggests that MSC-derived exosomes (MSC-Exos), as cell-free mediators, retain many of the beneficial effects of MSCs while exhibiting improved safety and stability profiles. MSC-Exos carry diverse bioactive cargo, including nucleic acids, lipids, and proteins, and can modulate immune responses, promote tissue repair, and restore barrier integrity. In this review, we place particular emphasis on both immunoregulation and tissue barrier protection as dual mechanisms underlying MSC-Exos efficacy in GVHD. We further discuss emerging preclinical and clinical evidence, as well as key challenges in translation.

## 1. Introduction

GVHD is a major cause of morbidity and mortality following allogeneic hematopoietic cell transplantation (allo-HCT) and is commonly categorized as acute (aGVHD) or chronic (cGVHD). Classic aGVHD typically develops within the first 100 days and involves inflammatory injury of the skin, gastrointestinal tract, and liver, presenting with erythematous rash, cholestatic hepatitis, nausea/anorexia, abdominal pain, diarrhea, gastrointestinal bleeding, or ileus. Similar features can also occur beyond day 100, termed late-onset aGVHD. By contrast, cGVHD is characterized by persistent inflammation with fibrosis and sclerosis, affecting multiple organs, most frequently the skin and its appendages, oral cavity, liver, eyes, gastrointestinal tract, genitalia, fascia, and the musculoskeletal system [1,2,3,4]. GVHD pathogenesis is multifactorial. Donor lymphocytes recognize host antigens presented by dendritic cells (DCs) and macrophages, leading to activation of donor T and B cells and initiating an adaptive immune response. This immune response damages the recipient’s tissues, culminating in systemic inflammation, multi-organ injury, inflammatory infiltration, and cytokine release. Simultaneously, the damage-related molecular patterns (DAMPs), such as HMGB1, heat shock proteins (HSP), and mitochondrial DNA, are released from mechanical damage, ischemic shock, and reperfusion injury caused by organ transplantation and tissue damage during aGVHD. The pathogen-related molecular patterns (PAMPs) are produced by dead pathogens (bacteria, fungi) during aGVHD. These two patterns directly activate macrophages and DCs via signals and engage pattern-recognition receptors (TLRs, NLRs, and cGAS) and their downstream pathways. Activated innate immune cells express and release high levels of proinflammatory cytokines (including IFN-α, IL-6, and TNF-α) and promote antigen presentation, thereby driving activation of T and B cells, cytotoxic effector programmes, and, ultimately, acute and chronic tissue injury that compromises both survival and quality of life [1,5]. Multiple agents and cellular therapies have been explored for aGVHD, including glucocorticoids, JAK1/2 inhibitors, calcineurin inhibitors, rapamycin (an mTOR inhibitor), antibodies targeting lymphocytes, inflammatory cytokines, or co-stimulatory pathways, as well as regulatory T-cell (Treg) or MSC-based therapies[6,7,8]. Nevertheless, systemic glucocorticoids remain the standard first-line treatment for aGVHD and cGVHD [1,3,4], despite severe side effects with prolonged use and limited efficacy, and approximately 50% of patients develop drug resistance [9,10]. Therefore, novel, safe, and effective immunomodulatory strategies for GVHD are urgently needed.

Exosomes (Exos) are pivotal mediators of intercellular communication. As nanoscale extracellular vesicles (EVs) carry proteins, nucleic acids, and lipids, they have emerged as versatile platforms for immune regulation and tissue repair [11,12]. Exo can cross physiological barriers and deliver bioactive cargo to recipient cells, thereby shaping cellular function and contributing to the initiation, maintenance, and resolution of inflammation across disease stages [13]. Early clinical and preclinical studies demonstrated that infusion of MSCs could ameliorate steroid-refractory GVHD by suppressing donor T-cell activation, inflammatory cytokine production, and tissue-repair ability [14,15,16], thereby providing a biological rationale for exploring MSC-derived exosomes (MSC-Exos) as a cell-free therapeutic alternative [17,18]. Studies suggest that MSC-Exos alleviates GVHD and improves survival by restraining T-cell expansion, promoting Treg differentiation, and reducing pro-inflammatory cytokine production [19,20]. In this review, we summarize the latest research progress and clinical prospects of MSC-Exos in GVHD, providing a framework for future development of GVHD immunotherapies. However, despite promising results, the use of MSCs remains limited by concerns regarding their in vivo behavior, including potential ectopic tissue formation [21,22], pro-tumorigenic effects [23,24], and heterogeneity in therapeutic efficacy [25,26,27]. In recent years, attention has shifted toward MSC-derived exosomes (MSC-Exos), which mediate intercellular communication through transfer of bioactive cargo and may recapitulate many of the immunomodulatory functions of parental MSCs while avoiding risks associated with live-cell therapies [17,18].

## 2. Exos Structure and Immunomodulatory Functions

### 2.1. Exos Biogenesis and Composition

Exos are a subclass of extracellular vesicles (EVs) measuring ~30–150 nm. They originate from multivesicular bodies (MVBs) formed within the endosomes. Their formation involves both ESCRT-dependent or ESCRT-independent pathways for endosomal transport. Exos Biogenesis and secretion are controlled by Rab GTPases (Rab27a/b, Rab11, Rab35) and autophagy-related proteins (Atg5, Atg16L1) [28]. Fusion of MVBs with the plasma membrane releases intraluminal vesicles as Exos [29,30]. Exos is enclosed by a lipid bilayer enriched in cholesterol, sphingomyelin, and phosphatidylserine (PS), with raft-like domains that include Flotillin-1. This membrane stabilizes and protects their cargo in the extracellular space. The cell membrane surface is equipped with characteristic proteins, including CD9, CD63, and CD81 membrane surface molecules, ESCRT-related proteins such as TSG101 and Alix, as well as HSP70 and HSP90, etc. The high degree of bioactivity and regulatory functions of Exos derives from diverse cargoes, such as proteins (enzymes, signaling molecules, and cytoskeleton components), lipids (various lipids and metabolic intermediates), and nucleic acids (miRNAs, lncRNAs, circRNAs, mRNAs, and DNA fragments) [31,32].

### 2.2. Core Functions of Exos

In producer cells, Exos may support homeostasis by exporting excess or unwanted components, although this remains incompletely defined. In recipient cells, they are key vehicles for intercellular communication. Exos release can be actively regulated and cargo-selective. Transferred proteins, lipids, and nucleic acids reshape signaling across cells and organs, tune immunity, modulate host–pathogen interactions, and influence tumor progression, cardiovascular, and CNS disorders, as well as tissue repair [33,34,35]. Exos can also affect pharmacokinetics, potentially improving bioavailability while reducing toxicity [31,32]. Accordingly, native or engineered Exos are being developed as nanocarriers for shRNA, siRNA, chemotherapeutics, and immunomodulators [36,37]. Furthermore, Exos-carried molecules are also promising liquid-biopsy analytes for disease diagnosis [13,38].

#### 2.2.1. Uptake Routes and Signaling Consequences

Exos enters cells through multiple routes. One is membrane fusion, potentially facilitated by tetraspanin complexes or integrins, although details remain unresolved [39]. A second route is phagocytosis via IgFc, complement or scavenger receptors, requiring actin, PI3K, and dynamin 2 [30]. This route also contributes to Exos clearance. The macropinocytosis process is driven by actin and involves the invagination of the intracellular membrane and non-specifically uptakes in a large amount of extracellular soluble substances, including Exo, etc. Many proteins are involved in the regulation of macropinocytosis. It has been found that the mutant K-RAS protein in tumors has a stronger driving effect than the wild-type K-RAS protein, increasing uptake of extracellular material (including Exos) and supporting tumor metabolism [40,41]. Moreover, PI3K and sodium ions modulate the process of micropinocytosis. Exos is also internalized by endocytosis, including clathrin-dependent receptor-mediated pathways and lipid-raft/caveolae routes involving caveolin-1 (Cav-1) or Cav-1–independent mechanisms driven by RhoA, CDC42, and ARF6. In addition, the receptor cells can also obtain signals through soluble substances transduced by Exos released by the host cells, as well as via paracrine methods [42].

After uptake, exosomal proteins, nucleic acids, and lipids regulate recipient-cell phenotypes. For example, pancreatic ductal adenocarcinoma Exos was reported to be enriched in the macrophage migration inhibitory factor (MIF). Kupffer cells captured the Exos from pancreatic ductal carcinoma, which was injected into the spleen. MIF promotes Kupffer cells to increase release of TGF-β, which subsequently activates hepatic stellate cells and significantly upregulates fibronectin (FN) expression. It recruits bone marrow myeloid cells to the liver and differentiates them into macrophages and neutrophils, thereby forming a fibrotic microenvironment—the pre-tumor metastasis microenvironment, promoting the local colonization of pancreatic cancer cells in the liver, and further forming liver metastases [43]. Exosomal nucleic acids can be equally instructive. MiR-150, rich Exos from THP-1 cells, or atherosclerosis plasma transferred miR-150 to HMEC-1 cells, suppressed c-Myb and enhanced migration, implicating Exos in atherogenesis [44].

#### 2.2.2. Immune Regulation

Exos transmits immunoregulatory cues that reshape T cells, DCs, natural killer (NK) cells, macrophages, and neutrophils, thereby coordinating inflammation, tolerance, and repair. Ji and his colleagues recently discovered that thymic stromal lymphopoietin (TSLP) treated DCs-derived Exos (DC-Exos) contain high levels of miR-21. This miR-21 reduced Smad7 in CD4^+^ T cells, increased RORγt, decreased Foxp3, and skewed differentiation towards Th17 at the expense of Treg, amplifying IL-17–driven inflammation [45]. However, in asthma, elevated OX40L and IL-4 with reduced IFN-γ suggest a Th2-biased milieu. Consistently, TSLP-activated DC-Exos enriched in OX40L promoted Th2 differentiation and increased IL-4, supporting asthma pathogenesis [46]. Radiotherapy can convert “cold” tumors into “hot” tumors, enhancing CD8^+^ T-cell immunity, but the mediators were unclear. Tumor Exos from irradiated breast cancer cells transferred dsDNA to DCs, activated STING signaling, increased co-stimulation and IFN-β, and triggered tumor-specific CD8^+^ T-cell responses [47]. In sepsis models, miR-127-5p from MSC-Exos reduced lung injury via targeting CD64 to suppress NET formation and inflammatory cytokines [48]. NK-92 Exos induced by IL-15 and IL-21 were enriched in granzymes B and H and displayed potent tumor cytotoxicity. However, loss-of-function studies suggested granzymes were not the sole drivers. Instead, IL-15 plus IL-21 increased exosomal CD226 (DNAM-1) and CD226 blockade with antibody reduced cytolysis [49]. Sepsis plasma EVs activated microglia and induced dose-dependent secretion of CXCL2 and IL-6 [50]. Intracerebroventricular delivery of the EVs drove recruitment of intracranial innate immune cells, including monocytes and neutrophils, and elevated cytokine expression significantly, precipitating neuroinflammation. Neurons were not directly affected by EVs in vitro, but conditioned medium from EV-stimulated microglia induced neuronal death. Inhibiting EV miRNAs (miR-146a-5p, miR-122-5p, miR-34a-5p, and miR-145a-5p) partially reversed CXCL2 induction, implicating a TLR7–MyD88 axis [51].

## 3. MSC-Exos: Functions and Clinical Development

### 3.1. Clinical Momentum of MSCs Therapy and Remaining Challenges

MSCs are widely distributed and readily accessible. They exhibit self-renewal and multilineage potential and show low immunogenicity (low MHC class I and minimal MHC class II expression) with robust and relatively stable immunosuppressive activity [52]. These features have driven broad exploration in GVHD, tissue repair, autoimmune diseases, Alzheimer’s disease, and ageing-related indications [53]. Early investigator-initiated studies supported clinical activity of MSCs in autoimmune diseases such as lupus [54], followed by phase I–III trials internationally with encouraging outcomes [55]. In late 2024, the first MSCs-based product received US FDA approval for steroid-refractory GVHD, marking a milestone for MSC therapeutics [56]. Updated consensus definitions and reporting standards from the International Society for Cell & Gene Therapy (ISCT)—including guidance for MSC-based products [57] and minimum peer-review criteria for MSCs clinical trials in autoimmune disease—should further accelerate clinical translation [58].

However, safety concerns persist. Prolonged passaging can introduce genetic alterations or mutations and senescence, raising theoretical risks of tumor development [59], particularly with late-passage MSCs. Generally, it is believed that cells remain safe within the first eight generations. However, starting from the ninth generation, cells begin to show signs of aging. Genetic sequencing has revealed that this process begins from the fifth generation and accumulates significantly by the ninth generation [60]. Therefore, injecting aging MSCs into the body may pose a potential risk of tumor formation. Even non-transformed MSCs may home to tumors, integrate into the tumor microenvironment, and support proliferation, migration, invasion, and therapy resistance via cytokines, Exos, and metabolic reprogramming [61]. Additional limitations include organ entrapment, microthrombosis, and host rejection [62]. These constraints have intensified interest in MSC-Exos as a cell-free strategy for GVHD prevention and treatment.

### 3.2. Advantages, Mechanisms, and Clinical Applications of MSC-Exos

#### 3.2.1. Advantages and Biological Activities of MSC-Exos

Compared with live MSCs, MSC-Exos offers several practical and biological advantages. (i) Free tumorigenicity: MSC-Exos lacks replicative capacity, which substantially reduces the risk of uncontrolled proliferation or ectopic tissue formation associated with cell-based therapies. However, it should be noted that MSCs themselves have been reported to contribute to ectopic tissue formation [21,22] or tumor-supportive microenvironments under certain conditions [63]. (ii) Low immunogenicity: With minimal MHC class I/II expression, MSC-Exos limits immune recognition and clearance, supporting allogeneic use without stringent HLA matching [64,65]. (iii) Multifunctional cargos: MSC-Exos carries cytokines (VEGF, EGF, TGF-β, and IL-8), RNAs (mRNA, miRNA, lncRNA, and circRNA), enzymes, signaling proteins, and membrane lipids. Together, these components readjust gene expression and signaling to mediate anti-inflammatory, anti-apoptotic, pro-proliferative, pro-differentiation, pro-angiogenic, and immunoregulatory effects [66]. (iv) Barrier penetration: Their nanoscale size facilitates tissue penetration and delivery across biological barriers, which may be particularly advantageous for targeting inflamed organs in GVHD [66]. (v) Greater stability and druggability: MSC-Exos better tolerates environmental stress than MSCs and is more amenable to purification, standardization, storage, and transport, facilitating clinical manufacture. (vi) Scalable sourcing: MSCs can be isolated from bone marrow, adipose tissue, umbilical cord, and placenta, enabling relatively scalable, low-ethical-burden production of MSC-Exos [66].

#### 3.2.2. Clinical Applications of MSC-Exos

These properties have motivated broad clinical exploration of MSC-Exos. (i) Tissue repair and regeneration: MSC-Exos supports bone and cartilage repair, accelerating wound healing with reduced scarring [67]. It can also promote the proliferation, migration, and angiogenesis of skin cells, accelerate the healing of difficult-to-heal wounds such as burns and diabetic foot ulcers, and reduce scar formation [62]. In addition, they deliver trophic factors/miRNAs that promote neuronal survival and axonal regeneration while limiting glial scarring [68]. They also protect cardiomyocytes after myocardial infarction, reduce apoptosis, enhance angiogenesis, and improve cardiac function [66]. (ii) Immunomodulation and anti-inflammation: MSC-Exos restrains excessive T- and B-cell activation and dampens macrophages/DC effector functions, thereby reducing pathological immune responses. They are being explored in rheumatoid arthritis, systemic lupus erythematosus, and multiple sclerosis [69]. (iii) Broader disease indications: Beyond cardiac repair, MSC-Exos may improve endothelial function and suppress inflammation/oxidative stress in pulmonary arterial hypertension and atherosclerosis. In liver disease, they can inhibit stellate-cell activation, limit injury, and support regeneration [70]. In kidney disease, they reduce inflammation and apoptosis while promoting tubular repair [66]. Ocular delivery (intraocular injection or eye drops) has been explored for retinal degeneration, corneal injury, and dry eye [71]. (iv) Targeted delivery platforms: With intrinsic (and engineerable) tropism and high biocompatibility, MSC-Exos is attractive carriers for chemotherapeutics and nucleic-acid payloads (including siRNA and CRISPR–Cas9), enabling more precise targeting (for example, to tumor cells) while reducing off-target toxicity [72].

## 4. MSC-Exos in Prevention and Treatment of GVHD: Mechanisms, Clinical Potential, and Challenges

GVHD is a leading cause of mortality after allo-HSCT and has historically been managed primarily with systemic glucocorticoids [2,3]. As the biology and immunomodulatory functions of MSC-Exos have become clearer, their potential in GVHD prevention and therapy has gained increasing attention [2,3].

### 4.1. MSC-Exos Reshape Immune Responses

#### 4.1.1. MSC-Exos Limit DC Maturation and Function

DCs bridge innate and adaptive immunity and are key initiators of adaptive immune responses. In GVHD, host antigen-presenting cells (APCs), particularly DCs, present host antigens to donor T cells (directly or indirectly), triggering TCR signaling, effector T-cell (Teff) differentiation, and production of pro-inflammatory cytokines such as IFN-γ and TNF-α. These responses drive tissue damage in major target organs, including skin, liver, and gut [73]. MSC-Exos-treated DCs show reduced expression of CD40, CD80, CD86 [19], and MHC-II [74]. Under LPS-driven maturation, MSC-Exos can increase indoleamine 2,3-dioxygenase (IDO), thereby constraining DC maturation. MSC-Exos-conditioned DCs prolong graft survival in murine skin-transplant models [74]. They also shift DC cytokine output (lower IL-6 and IL-12p70, higher IL-10), consistent with attenuated maturation and antigen-presenting capacity [19]. In GVHD mouse models, Li and colleagues reported that human bone marrow MSC-Exos increased splenic tolerogenic DC subsets (CD8α^+^ cDCs and CD11b^+^ cDCs) by ~7–8-fold compared with human fibroblast-derived exosomes (Fib-Exos) [75].

#### 4.1.2. MSC-Exos Dampen Monocyte–Macrophage Activation and Promote M2 Polarization

Macrophages exhibit remarkable functional plasticity and can polarize into distinct phenotypic states in response to microenvironmental cues, with the classically activated M1 and alternatively activated M2 phenotypes representing two extremes of a dynamic spectrum [76]. M1 macrophages are typically induced by TNF-α, LPS [77] only or plus with IFN-γ [78] and GM-CSF [79]. Activation of pattern recognition receptors, particularly TLRs, triggers downstream signaling cascades such as the NF-κB pathway [80], leading to the robust production of pro-inflammatory factors, including iNOS, TNF-α, IL-1β, and IL-6 [80,81]. Functionally, M1 macrophages exhibit potent antimicrobial and cytotoxic activity through the generation of ROS and NO, thereby amplifying inflammatory responses and promoting pathogen clearance. They are further characterized by high expression of co-stimulatory and Fc receptor molecules, such as CD80, CD86, CD64, CD16, and CD32, as well as enhanced antigen processing and presentation capacity, which supports T cell activation. In contrast, M2 macrophages are induced by IL-4 and IL-13 and are associated with anti-inflammatory and tissue-repair functions. They exert inhibitory effects on immune inflammation by secreting anti-inflammatory factors such as IL-10 and TGF-β, promoting tissue repair and angiogenesis. M2 macrophages are characterized by expression of markers such as CD206, arginase-1 (Arg-1), CD163, and Dectin-1. In the tumor microenvironment, they promote tumor progression by producing VEGF and CCL22, which recruit Tregs and suppress anti-tumor immunity.

Importantly, MSC-Exos has emerged as key regulators of macrophage polarization. In the preclinical model studies, it has been demonstrated that MSC-Exos suppresses M1 activated by LPS while promoting M2 polarization [82]. In vitro (RAW264.7 [19,83]) and in vivo (peritoneal macrophages [19]), MSC-Exos increased CD206 [19,83] and Arg-1 [83] while reducing M1-like features [19,83] (including CD86 [83]), consistent with a shift towards an M2-like programme that may alleviate aGVHD-associated inflammation and tissue injury. It has been demonstrated that umbilical cord–derived MSC exosomes (UCMSC-Exos) ameliorate steroid-resistant asthma in an ovalbumin (OVA)-induced animal model by modulating immune responses. UCMSC-Exos promoted macrophage polarization from the pro-inflammatory M1 to the anti-inflammatory M2 phenotype, as evidenced by decreased mRNA and protein levels of iNOS and CD86, alongside increased expression of Arg1 and CD206, both in vivo and in vitro. Mechanistically, these effects were mediated through inhibition of TRAF1 expression and suppression of NF-κB and PI3K/AKT-signaling pathways [84]. In a skin-transplant cGVHD model, Guo and his colleagues showed that MSC-EVs reduced infiltration of CD11b^+^ monocytes and CD11b^+^F4/80^+^ macrophages in skin and decreased splenic myeloid accumulation while lowering the M1 fraction and favouring M2 polarization [85]. Zhou et al. also reported that MSC-Exos reduced corneal infiltration of CD11b^+^ macrophages in GVHD patients and lowered pro-inflammatory signals (IL-6, IL-1β, IL-17A, and CD86) in cornea and conjunctiva. Mechanistically, MSC-Exos deliver regulatory miRNAs, such as miR-146a [86], let-7b [82], and miR-21 [87], which target signaling intermediates (e.g., TRAF6, TLR4) and inhibit NF-κB signaling, thereby reprogramming macrophages toward an anti-inflammatory phenotype. Moreover, miR-204 from MSC-Exos was reported to target IL-6R, suppress the IL-6–IL-6R–STAT3 axis, and promote M1-to-M2 reprogramming, with therapeutic benefit in GVHD-associated dry eye disease [83]. In addition, a clinical study by Harrell et al. demonstrated that a novel MSC-derived product, termed Exosome-derived Multiple Allogeneic Protein Paracrine Signaling (Exo-d-MAPPS), enriched in immunomodulatory factors such as soluble TNFR I/II, IL-1R antagonist, and soluble receptor for advanced glycation end products, effectively attenuates chronic airway inflammation. Mechanistically, it suppresses the production of pro-inflammatory cytokines (TNF-α, IL-1β, IL-12, and IFN-γ) from lung-infiltrating macrophages, neutrophils, NK cells, and NKT cells while promoting the expansion of immunoregulatory cell populations, including M2 macrophages, tolerogenic DCs, and Tregs, accompanied by increased secretion of IL-10 [88].

#### 4.1.3. MSC-Exos Reprogramme T-Cell Responses

T-cell-mediated immune response is a primary driver of GVHD pathogenesis. A substantial body of preclinical studies indicates that MSC-Exos can reshape T-cell activation, differentiation, and effector function. It has been demonstrated that MSC-EVs inhibit the proliferation and division of T cells [89]. Moreover, BM-MSC-EVs also inhibited T cell proliferation and shifted to Teff activated by CD3/CD28-stimulation and preserved Treg in a GVHD mice model [90]. Liu et al. reported MSC-Exos carries immunoregulatory miRNAs, including miR-223, miR-204, and miR-16-5p. The miR-223 can suppress ICAM-1, limiting Teff activation, adhesion, and trafficking to spleen, liver, and gut, reducing IFN-γ/TNF-α/IL-17 output and improving survival in aGVHD models [91]. UCMSC-Exos was reported to correct redox–metabolic dysregulation in CD4^+^ T cells and suppress inflammatory cytokines, exosomal miR-16-5p targeted the ATF6/CHOP pathway to reduce ER stress and apoptosis, promote CD4^+^IL-10^+^ Treg differentiation, and mitigate splenic, hepatic, and intestinal injury [92]. In GVHD mice, MSC-Exos reduced circulating CD4^+^ and CD8^+^ T-cell numbers, proliferation, and CD8^+^ cytotoxicity, decreased Th17 cells, increased Tregs, suppressed IL-2/TNF-α/IFN-γ while increased IL-10 and prolonged survival [75]. Jiang et al. reported MSC-Exos also reduced CD4^+^/CD8^+^ activation, inhibited Th1 polarization and TNF-α producing, promoted Th2 and IL-5 releasing and Treg differentiation, and dampened inflammation. Importantly, some studies suggest preservation of GVL activity [19]. Not all findings are concordant. Trapani et al. reported minimal changes in total CD3^+^ T cells and a modest increase in CD4^+^ T cells after exposure to MSC-Exos, regardless of IFN-γ/TNF-α priming. Interestingly, primed MSC-Exos co-cultured with resting MSCs could transfer or induce a T-cell suppressive phenotype in the MSCs themselves [89]. In cGVHD, MSC-EVs were reported to reduce splenic CD4^+^CXCR5^+^PD-1^+^ Tfh frequencies [85]. Furthermore, MSC-Exos or MSC-EVs suppress T-cell activation not only indirectly via inhibition of APCs maturation[93] but also directly by transferring regulatory molecules that induce cell arrest via upregulation of p27kip1 protein and downregulation of Cdk2 protein [94] and metabolic reprogramming in effector T cells [95]. They reduce inflammatory cytokines and promote Treg expansion and immunosuppressive cytokine IL-10 [96]. Collectively, MSC-Exos tends to suppress Th1 and CD8^+^ Teff programmes while favouring Th2 and Treg differentiation, thereby improving survival in GVHD models.

#### 4.1.4. Effects of MSC-Exos on B-Cell Immunity

Trapani et al. reported that MSC-Exos suppress B-cell proliferation, with similar effects observed for resting versus IFN-γ/TNF-α-primed MSC-Exos [89]. Guo et al. observed increased total splenic CD19^+^ B cells after MSC-EVs treatment but reduced CD19^+^Fas^+^GL7^+^ germinal-center/follicular B-cell populations [85].

#### 4.1.5. MSC-Exos Modulate NK-Cell Activity

NK cells can influence GVHD outcomes, although context-dependent effects remain under active investigation. Exosomal TGF-β from fetal liver–derived MSC-Exos was reported to activate TGF-β–SMAD2/3 signaling in NK cells, suppressing IL-2-driven activation and proliferation, reducing NKG2D/NKp30/CD107a expression, and limiting cytotoxicity [97]. Both resting and primed MSC-Exos have been reported to inhibit NK-cell proliferation [89]. Historically, donor NK-cell activation has been linked to GVHD, yet other studies suggest NK cells can suppress GVHD while preserving GLV effects, implying a potential GVHD-GVL dissociation [98]. MSCs and MSC-Exos are broadly immunosuppressive [89] but may not abrogate GVL in some settings [19]. Thus, NK-cell roles in GVHD versus GVL remain incompletely defined, and the impact of MSC-Exos on GVL during GVHD requires further mechanistic study.

**Figure 1:**
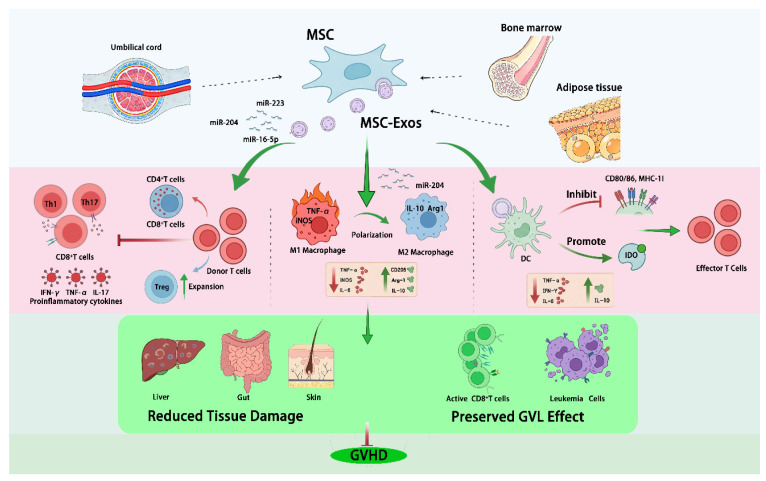
Mechanism of MSC-Exos-based immunotherapy in anti-GVHD.

Overall, MSC-Exos can deliver protein and nucleic-acid cargo that limits donor T-cell activation, expansion, and trafficking to target organs, reduces inflammation, restrains B-cell and NK-cell activity, expands Tregs, and promotes immune tolerance, supporting their therapeutic promise in GVHD (Figure 1).

#### 4.1.6. MSC-Exos Restoration of Intestinal Barrier Integrity

Beyond immune modulation, MSC-Exos may play a critical role in restoring intestinal epithelial barrier integrity in aGVHD. Evidence demonstrates that MSC-Exos promote epithelial cell proliferation, inhibit apoptosis, and enhance migratory capacity, thereby facilitating mucosal repair, as observed in dextran sodium sulfate (DSS)-induced inflammatory bowel disease (IBD) models [99]. In addition, MSC-Exos enriched with circHECTD1 has been shown to upregulate tight junction proteins, including ZO-1 and occludin, resulting in reduced intestinal permeability in ulcerative colitis (UC) models [100].

Taken together, these effects preserve epithelial barrier function, thereby limiting microbial translocation and subsequent activation of inflammatory cascades, which are key drivers of GVHD progression [101].

### 4.2. Progress in MSC-Exos Therapy for GVHD

As the research on MSC-Exo progresses, case reports have suggested clinical benefit, and broader translational efforts are emerging, positioning MSC-Exos as a frontier strategy for GVHD prevention and treatment.

#### 4.2.1. Broad Preclinical Promise of MSC-Exos in GVHD

Preclinical studies consistently demonstrate that MSC-Exos alleviates GVHD severity by suppressing T-cell activation, promoting regulatory immune subsets and reducing the production of pro-inflammatory cytokines. Mechanistic insights from in vitro and in vivo studies further support this immunomodulatory capacity. Fujii’s preclinical studies have declared that the human bone marrow MSC-EVs suppress anti-CD3/anti-CD28-driven PBMC expansion in vitro, including CD4^+^ and CD8^+^ T cells, while sparing naïve Tregs and preserving the proportions of B cells, NK cells, and mature myeloid cells. In vivo, systemic administration of MSC-EVs limited conversion of CD62L^+^CD44^–^- naïve T cells into CD62L^–^CD44^+^ Teff, expanded CD4^+^CD25^+^Foxp3^+^ Tregs, and partially mitigated disease progression in non–T-cell-depleted GVHD models. These changes were associated with reduced weight loss and decreased inflammatory infiltration in the liver, small intestine, and skin, as well as improved clinical scores and prolonged survival in aGVHD models [90]. Consistently, MSC-Exos enhances Tregs generation via APC-dependent mechanisms and improves survival in GVHD models [102]. In parallel, they significantly reduce Th17 responses while promoting Treg differentiation, thereby improving survival and reducing fibrosis in cGVHD models [103]. Further supporting these findings, Li and his colleagues reported reduced T-cell activation and lower IL-2, IFN-γ, and TNF-α, alongside increased anti-inflammatory cytokines such as IL-10 [75]. In addition, MSC-Exos has been shown to modulate DC function and promote Treg differentiation [19]. Across multiple independent preclinical models, MSC-Exos reduced clinical GVHD scores and improved survival [104]. In skin-transplant cGVHD models, Guo et al. found that MSC-Exos reduced monocyte-macrophage accumulation in skin and spleen and improved clinical manifestations, including alopecia and desquamation. Mechanistically, these effects were primarily associated with suppression of the TGF-β-SMAD2 signaling pathway [85].

Collectively, these studies establish a coherent mechanistic framework in which MSC-Exos modulate T-cell responses, rebalance Th17/Treg homeostasis, and attenuate inflammatory tissue damage. Systematic analyses of preclinical studies further support that MSC-EVs improve survival, clinical indices, and histopathology in experimental GVHD [105]. However, their therapeutic efficacy varies considerably depending on MSC source and preconditioning strategies, indicating pronounced functional heterogeneity [106]. Proteomic profiling further reveals that bone marrow-, adipose- and umbilical cord-derived MSC-Exos differ in composition and bioactivity, with preferential effects on regeneration, immunomodulation, or tissue repair, respectively [107]. Importantly, variability in source, concentration, dose, route, and timing of administration, as well as recipient inflammatory status, complicates cross-study comparison and limits the predictability of in vivo efficacy. Thus, standardized manufacturing and potency calibration remain key translational bottlenecks [106]. One proposed solution (Collo et al.) is to define functional units using an in vitro Treg-induction assay and then apply dose-standardized systemic regimens in aGVHD models, thereby achieving more consistent and reproducible therapeutic efficacy [108]. The key therapeutic functions in GVHD models are summarized in Table 1, and the representative clinical development of MSC-Based therapies for GVHD is listed in Table 2.

#### 4.2.2. Early Clinical Development of MSC-Exosome-Based Therapies for GVHD

Early clinical observations further suggest that MSC-Exos are safe and may improve symptoms in patients with severe or refractory GVHD, although the available evidence remains limited to small cohorts and case reports. Preliminary clinical applications have reported reductions in inflammatory markers accompanied by clinical improvement without significant adverse events, supporting further clinical evaluation. However, compared with MSCs—whose safety and efficacy are supported by extensive clinical trials and commercialization—MSC-Exos remains at an early stage of clinical translation in GVHD [19]. Initial clinical reports indicate that MSC-derived exosomes could alleviate severe therapy-refractory GVHD, accompanied by reduced inflammatory cytokines and clinical improvement without major adverse effects [65]. This therapeutic potential is partly attributed to their low immunogenicity [15,109] and the absence of risks associated with live cell therapies, such as microembolism, fibrosis [110], ectopic engraftment [21,22], and uncontrolled proliferation [18]. Nevertheless, rigorous GVHD trials are still limited in number and scale.

To date, registered studies have focused on cGVHD-associated dry eye [111] and aGVHD (including placenta-derived MSCs products and MSC-EVs) [112] while also extending to broader indications beyond GVHD, including progressive multiple sclerosis [113], acute respiratory failure (phase I/II randomized, double-blind, placebo-controlled designs) [114], type 1 diabetes, acute stroke, post–type A aortic dissection repair multi-organ dysfunction, Alzheimer’s disease, and dystrophic epidermolysis bullosa [115]. Importantly, most of these trials are designed primarily to evaluate safety and preliminary signals of efficacy, with limited public disclosure of detailed cohorts and outcomes. Consistent with these observations, case reports describe symptomatic improvement and reduced inflammatory markers after human placental MSC-Exos in cGVHD, without obvious adverse events [116]. Additional early studies further suggest potential benefit in severe, refractory GVHD, including cytokine suppression, improvement of mucocutaneous injury, and steroid sparing [112]. However, well-powered, randomized controlled trials are still required to establish optimal dosing strategies, confirm safety profiles, and determine true clinical efficacy [113].

**Table 2:** Representative clinical development of MSC-Exosome-Based Therapies for GVHD.

Indication	Source	Reference
Severe therapy-refractory GVHD	MSC-derived exosomes	[65]
cGVHD	Human placental MSC-Exos	[116]
cGVHD-associated dry eye	Umbilical MSC-derived exosomes	[111]
aGVHD; Severe, refractory GVHD	Placenta-derived MSC products and MSC-EVs	[112]

#### 4.2.3. Key Challenges for Translation

To achieve successful clinical translation of MSC-Exos for GVHD treatment, several key challenges must be addressed. (i) Incomplete mechanistic understanding: heterogeneity in exosomal cargo across MSC sources complicates pathway attribution and contributes to variable therapeutic responses. (ii) Manufacturing standardization and product comparability: significant variability in yield, composition, and potency hampers reproducibility and functional equivalence across preparations [106]. (iii) Dose and delivery optimization: the optimal dose, administration schedule, and route are likely context-dependent, varying with disease stage and phenotype, and require systematic clinical validation [108].

In the specific context of GVHD, additional challenges arise, including defining the optimal timing of MSC-Exos administration relative to HCT, balancing immunosuppressive effects with preservation of GVL activity, and minimizing infection risk. Therapeutic efficacy is further influenced by interactions with concurrent immunosuppressive regimens and dosing strategies.

Moreover, organ-specific efficacy—particularly within the gastrointestinal tract, a primary GVHD target—remains insufficiently characterized, and persistent variability in MSC source, culture conditions, and exosome isolation methods continues to drive heterogeneity in biological activity [117] and therapeutic outcomes [118,119]. These limitations underscore the urgent need for standardized manufacturing protocols and robust potency assays.

Emerging bioengineering strategies, including cargo loading and surface modification of MSC-Exos, may enhance targeting efficiency and therapeutic consistency; however, challenges related to regulatory approval, large-scale production, and quality control remain to be resolved [120,121].

## 5. Conclusions

In summary, MSC-Exos delivers miRNAs, proteins, and other bioactive cargoes that recalibrate immune responses, suppress excessive donor-derived immune activation, and attenuate inflammation-driven tissue injury, thereby alleviating GVHD. Notably, emerging evidence suggests that GVL activity may be preserved despite immunosuppression. Beyond immunomodulation, MSC-Exos also contributes to the restoration of epithelial barrier integrity, providing an additional mechanistic layer that is particularly relevant in aGVHD. However, clinical implementation remains constrained by limited standardization of manufacturing processes, challenges in ensuring product purity and potency, and the lack of robust frameworks for in vivo pharmacokinetics and safety assessment. In this context, bioengineering strategies for MSC-Exo are emerging as a promising approach to enhance therapeutic consistency and functional performance.

Overall, MSC-derived exosomes represent a highly promising, mechanism-informed therapeutic strategy for GVHD. Future efforts that integrate mechanistic insights with standardized manufacturing, technological innovation, and rigorous clinical evaluation will be critical to generating high-quality evidence and enabling precise, clinically effective MSC-Exos–based therapies.

## Figures and Tables

**Table 1:** Summary of Key Therapeutic Functions in GVHD models.

Therapeutic Functions	Molecular Mechanism	Key Outcome in GVHD Models	Reference
T-Cell Suppression	In vivo, systemic administration of MSC-EVs limited conversion of CD62L^+^CD44^–^ naïve T cells into CD62L^–^CD44^+^ Teff	Reduced weight loss, decreased inflammatory infiltration in liver, intestine, and skin; improved survival in aGVHD models	[90]
Rebalance Th17/Treg homeostasis	Reduce Th17 responses while promoting Treg differentiation	Enhanced immunosuppressive milieu, prolonged survival in aGVHD, reduced fibrosis in cGVHD models	[19,90,102,103]
Cytokine Modulation	Reduced T-cell activation and lower IL-2, IFN-γ and TNF-α, alongside increased anti-inflammatory cytokines such as IL-10	HBMSC-derived exosomes could attenuate aGVHD damage and promote the survival of aGVHD mice	[75]
DCs Modulation	MSCs-derived exosomes can notably inhibit the expression of costimulatory molecules and functional cytokine secretion of DCs	In animal HSCT models, MSCs-derived exosomes increase the survival rate of mice, and preserve the cytotoxic antileukemia effects of CD8^+^ T lymphocytes from recipient mice	[19]
Monocyte/Macrophage Regulation	A reduction in macrophage infiltration and associated with suppression of the TGF-β-SMAD2-signaling pathway.	Amelioration of alopecia and desquamation; attenuated dermal fibrosis in cGVHD	[85]

## Data Availability

No new data were created or analyzed in this study. Data sharing is not applicable to this article.
